# The Impact of the COVID-19 Pandemic in the Interrelationships Among Mental Health, Nutritional Status and Lifestyle Factors of Older Adults: A Cross-Sectional Study in the Pre- and Post-Covid Periods

**DOI:** 10.3390/nu17020249

**Published:** 2025-01-10

**Authors:** Antonios Dakanalis, Evmorfia Psara, Eleni Pavlidou, Sousana K. Papadopoulou, Georgios Antasouras, Gavriela Voulgaridou, Rena I. Kosti, Theophanis Vorvolakos, Maria Mentzelou, Apostolia Ntovoli, Maria Chrysafi, Odysseas Androutsos, Constantina Jacovides, Aspasia Serdari, Constantinos Giaginis

**Affiliations:** 1 Department of Mental Health, Fondazione IRCSS San Gerardo dei Tintori, Via G.B. Pergolesi 33, 20900 Monza, Italy; antonios.dakanalis@unimib.it; 2 Department of Medicine and Surgery, University of Milano Bicocca, Via Cadore 38, 20900 Monza, Italy; 3 Department of Food Science and Nutrition, School of Environment, University of Aegean, 81400 Myrina, Lemnos, Greece; fnsd21013@fns.aegean.gr (E.P.); elen.p.pavl@gmail.com (E.P.); g.antasouras@gmail.com (G.A.); maria.mentzelou@hotmail.com (M.M.); m.chrisafi3@gmail.com (M.C.); con.jacovides@gmail.com (C.J.); 4 Department of Nutritional Sciences and Dietetics, School of Health Sciences, International Hellenic University, 57400 Thessaloniki, Greece; souzpapa@gmail.com (S.K.P.); gabivoulg@gmail.com (G.V.); 5 Department of Nutrition and Dietetics, School of Physical Education Sport Sciences and Dietetics, University of Thessaly, 42132 Trikala, Greece; renakosti@uth.gr (R.I.K.); oandroutsos@uth.gr (O.A.); 6 Department of Psychiatry, School of Medicine, Democritus University of Thrace, University Hospital of Alexandroupolis, 68100 Thrace, Greece; tvorvola@med.duth.gr (T.V.); aserdari@yahoo.com (A.S.); 7 Department of Physical Education and Sport Sciences, Frederick University, Limassol 3080, Cyprus; aero.na@frederick.ac.cy

**Keywords:** COVID-19 pandemic, depressive behavior, quality of life, cognition state, physical activity, nutritional status, older adults, elderly

## Abstract

Background/Objectives: The COVID-19 pandemic has led to detrimental effects on diverse aspects of the mental and physical health of the general population worldwide. The elderly are more susceptible to COVID-19 infection compared to younger age groups. In this aspect, the purpose of the current survey is to evaluate the effect of the COVID-19 pandemic on the interrelationships among the sociodemographic and anthropometric characteristics, depressive behavior, quality of life, cognition status, physical activity and nutritional status of older adults. Methods: The present study constitutes a comparative, cross-sectional study of 4162 older adults (mean age ± standard deviation: 72.13 ± 8.1 years and 75.22 ± 8.2 years in the pre- and post-COVID-19 periods, respectively, and a male/female ratio of almost 1:1). We used validated questionnaires to assess depression, cognition function, quality of life, physical activity and nutritional status of the elderly in the pre-Covid and post-Covid periods. Relevant questionnaires were also used for collecting sociodemographic data, while anthropometric data were measured using standard protocols. Results: The present study finds that the COVID-19 pandemic influenced, in an independent manner, residence location, smoking status, overweight/obesity and abdominal obesity, depressive behavior, quality of life, cognition behavior, physical activity levels and nutritional status of the elderly. The COVID-19 pandemic was also related to employment and living status as well as family economic status. Nevertheless, the above three relationships were insignificant in the multivariate analysis. Conclusions: The COVID-19 pandemic exerted deleterious effects on several aspects of the mental and physical health of the elderly, which appeared to strongly persist in the post-Covid period. Future prospective population-based and well-organized surveys should be conducted to establish whether there is a causality long-term effect of the COVID-19 pandemic on diverse aspects of the mental and physical health of the elderly.

## 1. Introduction

Worldwide humanity was affected by the coronavirus infection of 2019 (COVID-19) which was established as a pandemic in the first months of 2020 [1]. This horrible pandemic led healthcare systems to crash, with considerable damage to lives and high morbidity. The COVID-19 pandemic led to exceptional disturbances in the daily life activities of people [2.3]. It promoted a harmful effect on human mental wellbeing worldwide, which was mainly attributed to the fear, uncertainty and isolation individuals experienced [2,3]. Some people experienced various aspects of mental health disturbances, mainly those who had been susceptible to these prior to the pandemic [4]. In this context, substantial meta-analyses have revealed that the incidence of depressive behaviors was considerably elevated during the COVID-19 pandemic [5,6]. In addition, a meta-analysis survey conducted of 32 studies from 18 countries reported a virtually two-fold increase in older adults suffering from deteriorating cognition [7]. The aggravation or beginning of the behavioral and psychological symptoms of dementia were also noted in comparison with those of the elderly presenting healthy cognition, including people who experienced weaking cognition and deteriorating mental health [7]. Thus, this meta-analysis highlighted that an unhealthy lifestyle resulting from loneliness may contribute to deteriorating cognitive and mental health outcomes in the elderly [7].

In a large and nationally representative household panel study from Germany, anxiety and depression symptoms reduced, while loneliness increased, between the first and second COVID-19 waves [8]. However, depressive symptoms increased and the surge in loneliness was steeper in those with versus without clinically relevant depressive symptoms in 2019 or a history of a depressive disorder before the COVID-19 pandemic [8]. A nationally representative longitudinal study of Irish adults showed that considerably more people were diagnosed with depression in February 2019 than in March–April 2020 [9]. Moreover, Wechsler et al. suggested a dissociation between perceived changes in the subareas of stress and mental health, with a particular experience of rises in depression and general stress symptoms [10]. An Italian longitudinal study found that the COVID-19 emergency had substantial effects on the mental state of the population, with important repercussions for individuals and collective well-being during and probably also after the pandemic [11]. Another study in Germany also showed that the most elderly population seemed to present moderately stable mental health compared with a slight increase in symptomatology within the first year of the pandemic [12]. A study of Spanish adults provided novel evidence that although life has resumed a sense of normalcy after the COVID-19 pandemic, the mental health of key populations is still suffering, and further intervention and resources are needed [13]. Similarly, in an Estonian longitudinal study, latent profile analysis identified four distinct trajectories of change in stress and coping, involving resilient, stressed, recovering and deteriorating trends [14]. Participants belonging to the positively developing stress trajectories reported higher active leisure engagement compared to those with negatively developing stress trajectories [14]. In a longitudinal COVID-19 coping study spanning from April/May 2020 to April/May 2021 in older adults in the USA, some participants reported positive identity themes like rethinking and revising priorities and the realization of strength and resilience [15]. This study also indicated harmful effects, including identity disruption. Notably, individuals reporting identity disruption showed worse well-being at baseline and remained consistently worse over time [15]. However, studies exploring and comparing the incidence of depressive behavior prior to and after the COVID-19 pandemic, utilizing nationally representative data, remain scarce, especially for the elderly age group.

In the last few years, quality of life has mainly been investigated in research focusing on non-communicable and prolonged disorders. The health-related quality of life (HRQOL) questionnaire is well-recognized as a dynamic, independent and multi-dimensional questionnaire tool, including physical, social, psychological and environmental items [16]. Several surveys showed that the COVID-19 pandemic reduced the HRQOL score in children, adolescents, middle-aged adults and elderly people [17]. COVID-19 confinement negatively influenced cognitive function, especially for older adults [17]. This finding was related to higher levels of physical inactivity, restricted contact with basic services, loneliness and decreased and/or absent family and social support [18]. Throughout the COVID-19 universal health crisis, adults aged ≥60 years had a higher probability of depressive behavior, low or poor health-associated quality of life, and poor lifespan happiness status [19,20]. Reduced community networks and elevated community inaccessibility simultaneously acted as crucial mediators [21,22]. This fact expanded negative feelings and lowered lifespan happiness in this age group [21,22]. In addition, the COVID-19 pandemic negatively altered physical activity in approximately 50% of the people studied [23]. This led to a substantial rise in body weight, resulting in an elevated prevalence of people being overweight and obese [23]. The World Health Organization (WHO) has currently reported that 95% of COVID-19 deaths happened in older adults above 60 years, and more than half of all deaths occurred in elderly people aged over 80 years [24,25]. Significantly, 80% of the deaths exhibited one fundamental comorbidity, especially cardiovascular disorders, hypertension, diabetes and several other prolonged pathological states [24,25].

Several features were identified as influencing COVID-19 infection, like socioeconomic and lifestyle aspects [26]. The performance of self-control actions for infection can recognize the risk factors, influencing patients’ living habits and leading to reduction in diseases and death. In this aspect, a healthy nutritional status was recognized as a main factor in enhancing the action of the immune system against COVID-19 infection [27,28]. Moreover, during the COVID-19 pandemic, eating habits were adversely influenced by stressful behavior, distress and anger [29]. Thus, a high level of perceived distress was associated with unhealthy dietary patterns and poor diet quality [29]. Malnutrition has detrimentally affected pulmonary functions by resulting in a decrease in respiratory muscle strength, changing the ventilation capacity and leading to a deterioration in immune system function [30]. More importantly, malnutrition was found to increase mortality and morbidity in seriously ill patients [30]. Due to the COVID-19 restrictions, most people had serious difficulties adopting a healthy and well-adjusted diet program [28]. In support of this view, people usually chose to eat high-calorie accessible foodstuffs, snacks and junk food, as a replacement for fresh food, like fruits and vegetables [28]. Characteristically, in a recent multicenter prospective study, malnutrition impaired the clinical outcomes, increasing the morbidity and mortality of COVID-19 patients [31]. Notably, the COVID-19 quarantine exerted substantial impacts on nutritional behavior and physical activity, underlining a rise in food uptake and a decline in physical movement, which have further led to body weight increase [32]. Thus, most people, including the elderly, stayed at home for several hours, increasing the demand for easy foodstuffs to overcome the anxiety triggered by these outstanding conditions [33]. Additionally, the comfort of contact with food for the duration of the home restriction and changed emotional behaviors led to a rise in binge-eating disorder incidents in obese populations [34].

Few surveys have investigated the effect of COVID-19 pandemic on the interrelationships among sociodemographic and anthropometric factors, depressive behavior, quality of life, cognition state, physical activity and the nutritional status of the elderly, comparing the pre-COVID-19 period with the post-COVID-19 period. Moreover, there is no currently available data concerning the persistent changes caused by the COVID-19 pandemic in diverse characteristics of mental and physical health of older adults in their daily life during the post-Covid period. In this aspect, the purpose of the current survey was to assess the effect of the COVID-19 pandemic on the interrelationships among sociodemographic and anthropometric parameters, depression, quality of life, cognition state, physical activity and nutritional status of the elderly by comparing the pre-Covid period with the post-Covid period.

## 2. Materials and Methods

### 2.1. Study Population

**Figure 1:**
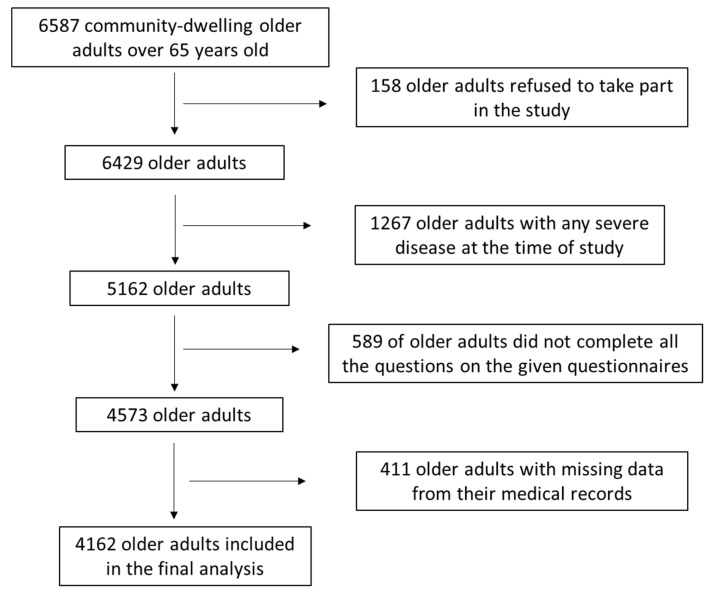
Flow chart diagram of study population enrolment.

Initially, 6587 community-dwelling adults aged above 65 years old were arbitrarily enrolled from 10 different Greek regions, including both urban and rural regions. The 10 different regions comprised Athens, Thessaloniki, Alexandroupoli, Larissa, Ioannina, Patra, Kalamata, Crete and the North and South Aegean. Enrollment to the survey was conducted between April 2018 and October 2019 in community-dwelling elderly people concerning the pre-COVID-19 period. The enrollment to the study for the post-COVID-19 period was begun after February 2022 and lasted until June 2023. The enrolled elderly people were mostly recruited when they visited hospitals for routine check-ups and in municipal care units in which entertainment events for the elderly took place. Several older adults were also enrolled during visits for nutritional support and counselling with nutritionists and dietitians from our research group, as well as during visits to nutritionists and dietitians who collaborated with our research group. A detailed depiction of the study enrolment as a flow chart diagram is shown in Figure 1. Among the 6587 community-dwelling older adults, 158 declined to take part in the study and 1267 were diagnosed with a severe disease during the study. Moreover, 589 of the initially recruited older adults did not complete all the questions on the given questionnaires, while for 411 participants there was missing data from their medical records. Finally, 4162 older adults were included in the final evaluation by utilizing the relevant exclusion and inclusion criteria, leading to a final response rate of 63.2%.

The present study was permitted by the Ethics Committee of the University of the Aegean (ethics approval code: 11/17.1.2018, date of approval: 17 January 2018). It was also in agreement with the World Health Organization (52nd WMA General Assembly, Edinburgh, Scotland, 2000). All the assigned older adults’ data remained private. All the enrolled older adults did not have any severe disorder at the time of the survey. Moreover, they were informed about the aim of the study and signed a consent form. Sample size estimation was accomplished using the PS: Power and Sample Size calculator program. Randomization was conducted by the use of an order of random binary numbers (i.e., 001110110, where 0 stated assignment and 1 not enrolment to the survey). The calculation of the power of our study population size found a power equal to 88.3%.

The present study is a comparative, cross-sectional survey that has evaluated sociodemographic parameters like age, gender, educational level, family economic status, employment, type of residence, living status and smoking habits in the pre-Covid and post-Covid periods. All sociodemographic data were collected by face-to-face meetings amongst the enrolled elderly and experienced staff to minimize recall bias. The educational level was classified into (a) primary education, (b) secondary education and (c) university studies. Financial status was estimated by the elderly people’s yearly salary, and it was categorized as 0 ≤ 5000 EUR, 1 ≤ 10,000 EUR, 2 ≤ 15,000 EUR, 3 ≤ 20,000 EUR, 4 ≤ 25,000 EUR and 5 ˃ 25,000 EUR, based on the per capita gross domestic product. We additionally grouped economic status as low for a yearly salary ≤10,000 EUR, moderate for a yearly salary ˃10,000 EUR and ≤20,000 EUR and high for a yearly salary ˃20,000 EU.

### 2.2. Study Design

Anthropometric parameters were measured by body weight and height and were evaluated at the time of meetings to estimate body mass index (BMI) both in the pre- and post-COVID-19 periods. The weight of the elderly people was assessed using a Seca scale [Seca, Hanover, MD, London, UK], without shoes, to the closest 100 g, and height was measured using a portable stadiometer (GIMA Stadiometer 27335, London, UK), with no shoes on, to the closest 0.1 cm. WHO guidelines were used to classify the enrolled elderly as being or normal weight, overweight or obese [35]. The waist circumference was determined at the midpoint between the lower margin of the last palpable ribs and the top of the iliac crest [36]. The hip circumference was determined across the widest portion of the buttocks, with the tape parallel to the floor [36]. The waist–hip ratio (WHR) was estimated by dividing the waist circumference by the hip circumference. The WHR is recognized as a better indicator of abdominal obesity than BMI, which more effectively determines the probability of developing cardiometabolic diseases like diabetes [36].

Five suitable and validated questionnaires were applied for evaluating the depressive behavior, health-related quality of life, cognitive status, physical activity levels and nutritional status of the enrolled elderly people. In addition, depressive behavior was evaluated by the use of the geriatric depression scale (GDS) questionnaire, which contains 30 items [37]. The Mini Mental State Examination (MMSE) questionnaire was utilized to evaluate the cognitive status of the assigned elderly [38]. The MMSE is effective as a screening tool for cognitive impairment in older, community-dwelling, hospitalized and institutionalized adults [38]. The health-related quality of life (HRQOL) was assessed using the Short Form Health- Survey (SF-36) questionnaire, which includes 36 items assessing health status on eight subscales [39].

Physical activity levels were determined by applying the International Physical Activity Questionnaire (IPAQ) [40]. In this worldwide questionnaire, the participants report how much exercise they engaged in during a typical week [40]. The Mini Nutritional Assessment (MNA), a validated questionnaire, was used for determining the nutritional status of the enrolled older adults [41,42]. This screening and assessment tool constitutes a consistent scale and clearly defined thresholds for the nutritional assessment of elderly [43,44]. Collectively, the self-reporting scales of GDS, MMSE, HRQOL and IPAQ have been thoroughly and adequately checked for their reliability, validity and internal consistency (Cronbach’s α > 0.80) [37,38,39,40,41,42,43,44].

All the questionnaires were completed by experienced staff (e.g., medical and nursing staff) as well as by nutritionists and dietitians in one-to-one meetings with the enrolled elderly to reduce recall biases. The experienced staff informed and explained in detail to the assigned elderly people all the questions included in the questionnaires to enhance the consistency and the validity of the answers. For a number of participants (n = 320), the questionnaires were additionally completed again two weeks later to test the obtained responses for their consistency and validity. Comprehensive and efficient explanations and presentation instructions of the questionnaires were carried out to minimize possible recall biases, enhancing the validity and the reliability of the participants’ responses.

### 2.3. Statistical Analysis

The Kolmogorov–Smirnov test was used to evaluate whether the continuous variables followed a normal distribution. Student’s *t*-test was used for the continuous variables, which followed the normal distribution. The Chi-square test was utilized for the categorical variables. The continuous variables were given as mean value ± standard deviation (SD) and the categorical variables as absolute or relative incidences. Multivariate binary logistic regression was used to evaluate whether the COVID-19 pandemic was associated in an independent manner with nutritional status, depression, quality of life, cognitive status and physical activity after adjusting for potential confounding factors. As confounding factors, we included all the assessed variables that could have had a confounding effect. Multivariate regression findings were presented as relative risk (RR) and 95% confidence intervals (CIs). Differences were recognized as significant at *p* < 0.05 and the 95% confidence interval. Statistica 10.0 software, Europe (Informer Technologies, Inc., Hamburg, Germany) was used for the statistical analysis of the study data.

## 3. Results

### 3.1. Comparative Analysis of the Sociodemographic and Anthropometric Parameters Between Pre-Covid Period and Post-COVID Period

The mean age ± standard deviation of the enrolled older adults was 72.13 ± 8.1 years and 75.22 ± 8.2 years in the pre- and post-COVID-19 periods, respectively, with a male/female ratio of almost 1:1. As far as the sociodemographic and anthropometric parameters in the pre- and post-COVID-19 periods is concerned, a significant increase in participants’ age was recorded (Table 1, *p* = 0.0001). The incidence of unemployed participants increased significantly in the post-COVID-19 period compared with the pre-COVID-19 period (Table 1, *p* = 0.0286). A significantly higher incidence of participants living in rural regions in the post-COVID-19 period was observed than in the pre-COVID-19 period (Table 1, *p* = 0.0187). The proportion of older adult participants living alone also significantly increased in the post-COVID-19 period in comparison with the pre-COVID-19 period (Table 1, *p* = 0.0087). The family economic status of the assigned elderly people was considerably reduced in the post-COVID-19 period compared with the pre-COVID-19 period (Table 1, *p* = 0.0108). A substantial increase in the prevalence of heavy smokers in older adults was observed in the post-COVID-19 period in comparison with the pre-COVID-19 period (Table 1, *p* = 0.0031). In contrast, the older adults’ educational level was not significantly different between the pre- and post-Covid periods.

The mean BMI of the assigned elderly people was significantly increased by more than one point in the post-COVID-19 period in comparison with the pre-COVID-19 period (Table 1, *p* = 0.0005). Accordingly, the incidence of being overweight and obese in the elderly was significantly increased in the post-COVID-19 period compared with the pre-COVID-19 period (Table 1, *p* = 0.0002). Remarkably, 23.2% and 7.3% of the assigned older adults were affected by being overweight and obese, respectively, in the pre-COVID-19 period, while these percentages were considerably increased to 30.0% for overweight participants and to 14.3% for obese participants in the post-Covid period (Table 1). The incidence of abdominal obesity, expressed by the WHR index, was also significantly increased in the post-COVID-19 in comparison with the pre-COVID-19 period (Table 1, *p* = 0.0123). Characteristically, 16.9% and 8.3% of the participants had a medium or high WHR in the pre-Covid period, respectively, while these proportions were significantly increased to 21.9% and 10.4% in the post-Covid period, respectively (Table 1).

### 3.2. Comparative Analysis of Nutritional Status, Depression, Health-Related Quality of Life, Cognitive Status and Physical Activity Levels Between Pre-Covid Period and Post-Covid Period

**Figure 2:**
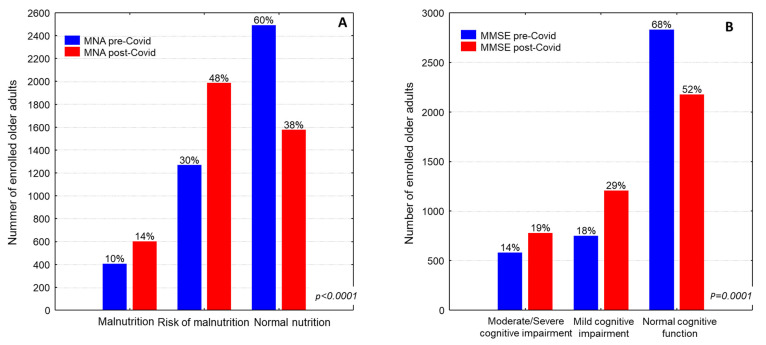
Comparative analysis of (**A**) nutritional status and (**B**) cognitive status between pre-Covid period and post-Covid period.

The mean nutritional status levels of the assigned elderly assessed by MNA was significantly lower in the post-COVID-19 period in comparison with the pre-COVID-19 period by more than one point (Table 1, 22.1 ± 4.5 vs. 23.8 ± 4.2, respectively, *p* = 0.0001). In the cross-tabulation, 30.5% and 9.7% of the assigned elderly were at risk of malnutrition or were malnourished, respectively, in the pre-COVID-19 period (Figure 2A, *p* = 0.0001). These proportions were significantly increased, reaching a prevalence of 48% for participants at risk of malnutrition and 14% at risk of being malnourished (Figure 2A)

Accordingly, the MMSE score was significantly reduced about two points in the post-Covid period in comparison with the pre-COVID-19 period (Table 1, 22.3 ± 5.2 vs. 24.4 ± 5.1, respectively, *p* < 0.0001). In fact, 18.0% and 14.0% were diagnosed with mild or moderate/severe cognitive impairment, respectively, in the pre-COVID-19 period, while these percentages were significantly increased to 29.0% and 18.7%, respectively, in the post-COVID-19 period (Figure 2B, *p* = 0.0001). This finding may be ascribed to the fact that the enrolled older adults were three years older in the post-Covid period than in the pre-Covid period.

**Figure 3:**
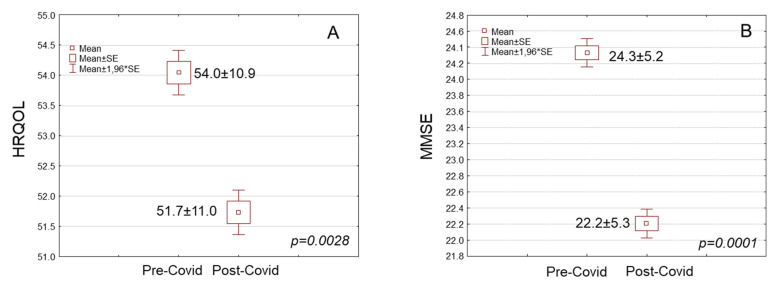
(**A**) Health-related quality of life (HRQOL) score and (**B**) Mini Menta State Examination (MMSE) score of the study population in pre- and post-Covid periods.

Participants’ GDS scores were significantly increased by more than two points in the post-COVID-19 period in comparison with the pre-Covid period (Table 1, 14.5 ± 4.3 vs. 12.2 ± 4.7, respectively, *p* < 0.0001). In the cross-tabulation, 30.3% of the older adults were found to have developed depressive symptomatology in the pre-COVID-19 period. This percentage was considerably increased to 46.5% in the post-Covid period (Table 1, *p* < 0.0001). The HRQOL score was considerably lowered by more than two points in the post-COVID-19 period in comparison with the pre-COVID-19 period (Figure 3A, Table 1, 51.7 ± 11.0 vs. 54.0 ± 10.9, *p* = 0.0028). The MMSE score was significantly lower, by more than two points, in the post-COVID-19 period in comparison with the pre-COVID-19 period (Figure 3B, Table 1, *p* = 0.0001). Moreover, the participants showed significantly lower physical activity levels in the post-COVID-19 period in comparison with the pre-COVID-19 period (Table 1, *p* = 0.0028). In the cross-tabulation, 39.4% of the participants showed low physical activity levels in the pre-COVID-19 period. This percentage was significantly elevated to 53.4% in the post-COVID-19 period (Table 1, *p* = 0.0008).

### 3.3. Multivariate Binary Logistic Regression Analysis Concerning Whether COVID-19 Pandemic Could Have an Independent Impact on Sociodemographic and Anthropometric Parameters and Lifestyle Factors when Comparing the Pre-COVID-19 Period with the Post-COVID-19 Period

In the multivariate binary logistic regression analysis, the COVID-19 pandemic had a significant independent effect on residence type, smoking behaviors, BMI and WHR status, depression, quality of life, cognition status, physical activity and nutritional status (Table 2, *p* < 0.05). The assigned elderly showed a 32% greater incidence of living in rural areas in the post-COVID-19 period in comparison with the pre-COVID-19 period (Table 2, *p* = 0.0347). The older adults also showed a 63% higher incidence of being heavy smokers in the post-COVID-19 period in comparison with the pre-COVID-19 period (Table 2, *p* = 0.0203).

The older adults also exhibited a 75% higher risk of being overweight or obese in the post-Covid period than in the pre-COVID-19 period (Table 2, *p* = 0.0148). In fact, the participants indicated an 85% higher risk of presenting abdominal obesity in the post-COVID-19 period than in the pre-COVID-19 period (Table 2, *p* = 0.0289). Employment status, living status and family economic status did not exert any considerable effect on the multivariate analysis results (Table 2, *p* ˃ 0.05).

The assigned elderly exhibited a two-fold greater probability of depression in the post-COVID-19 period in comparison with the pre-COVID-19 period (Table 2, *p* = 0.0001). Accordingly, the assigned participants exhibited a higher than two-fold likelihood of a worse HRQOL in the post-COVID-19 period than in the pre-COVID-19 period (Table 2, *p* = 0.0015). The older adult participants were also characterized by an 87% higher likelihood of presenting moderate or severe cognitive impairment in the post-COVID-19 period in comparison with the pre-COVID-19 period (Table 2, *p* = 0.0159). The assigned participants also showed a 65% greater probability of low physical activity levels in the post-COVID-19 period in comparison with the pre-COVID-19 period (Table 2, *p* = 0.0175). The enrolled participants also exhibited a more than two-fold greater risk of being malnourished in the post-Covid period in comparison with the pre-Covid period (Table 2, *p* = 0.0018).

## 4. Discussion

The present study constitutes one of the few cross-sectional surveys evaluating the impact of the COVID-19 pandemic on various aspects of the mental and physical health of the elderly, and also highlighting its potential effects on sociodemographic and anthropometric parameters, comparing the pre- and post-COVID-19 periods. The present survey suggested that the COVID-19 pandemic may affect residence type, smoking behaviors, BMI and WHR status, depression, quality of life, cognition status, physical activity and nutritional status after adjusting for several confounders. The COVID-19 pandemic was also associated with employment status, living status and family economic status in the unadjusted analysis. Nevertheless, the above relationships were not significant in the multivariate analysis. Thus, the COVID-19 pandemic may lead to several deleterious effects on older adults. These seem to persist, even if the effects of the COVID-19 pandemic have been considerably attenuated, at least for now.

The present survey showed that the elderly people exhibited a 32% greater prevalence of living in rural areas in the post-COVID-19 period in comparison with the pre-COVID-19 period. The above result is not surprising. In fact, several older adults had moved to the place they originally came from, which was mainly in rural areas, to avoid the COVID-19 restriction measures. In this aspect, it can be speculated that people in rural areas and small towns with a lower population density, lower connectivity and jobs less dependent on social interaction are usually exposed to COVID-19 to a lower extent [45,46]. Rural areas, villages and small towns were less affected by the COVID-19 lockdown as their populations could go outside without the worry of being in close contact with others [47,48,49]. In support of this view, several surveys showed that the prevalence of social isolation and loneliness was less common in rural regions than in urban regions, with a primary focus on loneliness [50]. Older rural residents frequently have family living close by [51,52]. Thus, it was more possible for these residents to have visitors throughout the pandemia compared to their urban counterparts [51,52]. Moreover, elevated levels of social media usage amongst rural-dwelling elderly people compared with those living in urban centers in the USA were reported [53]. Thus, some rural-dwelling elderly people were essentially prompt adopters of technologies to retain social and familial contact [54]. This fact further contributed to the connection of elderly people in rural regions throughout the pandemia, minimizing the probability of isolation and loneliness [54].

In our study, the elderly showed a 63% higher incidence of being heavy smokers in the post-COVID-19 period in comparison with the pre-COVID-19 period. In contrast, a recent Greek survey using an online questionnaire and including 200 smokers/vapers found that there was not any substantial difference in the everyday use of smoke/vaping throughout the lockdown measures [55]. However, this study was performed on a small sample size by using an online questionnaire, which decreases the validity and accuracy of the derived results [55]. On the contrary, a cross-sectional online survey conducted among 2511 young and middle-aged adults indicated that the subsequent emotional troubles resulted in higher smoking and worsened smoking behaviors amongst smokers throughout the COVID-19 quarantine [56]. In contrast, in a retrospective survey, a total of 9470 adolescents were evaluated, showing that there was no considerable change in the incidence of cigarette smoking prior to and throughout the COVID-19 pandemic amongst genders, age groups and type of education [57]. However, it was found that tobacco use throughout the COVID-19 pandemic was greater in susceptible groups like black adolescents as well as in those with psychological disorders [57].

Moreover, a meta-analysis of 77 clinical surveys found a rise in smoking behavior for most of the participants in 34 surveys [58]. However, 18 studies showed that a smoking decrease was the predominant response, while in the remaining 21 studies no changes in smoking habits were noted [58]. Thus, the existing evidence so far regarding the effect of the COVID-19 pandemic on smoking habits remains conflicting. In any case, it should be emphasized that smoking appeared to result in a worse prognosis in COVID-19 patients [59]. In addition, current or former smokers who were COVID-19 patients were susceptible to adverse hospital consequences and advanced COVID-19 progression [59]. A substantial prospective study including 402,978 participants showed that the relation of smoking with COVID-19 infection and subsequent death may be dependent on age [60]. Smokers and previous smokers aged under 69 years were at greater probability of COVID-19 infection [60]. When infected, older smokers had a two-fold higher risk of dying from COVID-19 compared to non-smokers, possibly facilitated by a higher likelihood of prolonged diseases [60].

COVID-19 confinement exerted harmful effects on health behaviors in the elderly in Europe. Nevertheless, the studies examining these effects on body weight gain and obesity in the elderly remain scarce. In a recent longitudinal study, during the COVID-19 pandemic, elderly people adopting unsafe health behaviors were more susceptible to present body weight gain and obesity, both in the short and long term [61]. The above study agrees with our results, which showed that elderly people exhibited a 75% higher risk of being overweight or obese in the post-Covid period. Moreover, our study revealed an 85% higher risk of abdominal obesity, which significantly increases the risk for several chronic diseases. Accordingly, a meta-analysis of 184 surveys involving 2,365,377 patients showed an enhanced incidence of being overweight and obese amongst adults aged more than 50 years, which substantially elevated the frequency of COVID-19 infections, severity and hospitalization [62]. A systematic review of 40 observational studies identified physical inactivity, a sedentary lifestyle and poor eating patterns throughout the COVID-19 pandemic as the most common risk factors for obesity [63]. Moreover, unhealthy foodstuffs consumption led to extreme behavioral stress, depressive and anxiety symptoms, and poor mood, especially in the elderly and ethnic minorities, which was shown to increase the risk of developing obesity throughout the COVID-19 pandemic [63].

In this aspect, the incidence of obesity among children and adolescents in Korea increased after the COVID-19 outbreak, which was related to a rise in the frequency of early comorbidities in adulthood [64]. Accordingly, a population-based study has also demonstrated that the prevalence of abdominal obesity was elevated amongst obese children and adolescents during the COVID-19 outbreak in Korea [65]. The above finding is in agreement with the elevated frequency of abdominal obesity that we found in our study in older adults, though. Several other studies showed that the COVID-19 confinement resulted in unhealthier nutritional habits, enhanced sedentary behaviors and reduced physical activity, resulting in excessive body weight, deregulation of glucose metabolism and higher metabolic risk in adults [66]. This study also showed that independent of any weight changes, individuals with type II diabetes experienced worsening glycemic and lipid parameters during the pandemic compared to those without type II diabetes [66]. A cross-sectional study reported that the COVID-19 confinement exerted harmful effects on the lifestyle (e.g., physical activity) and nutritional habits of Jordanian adults during the COVID-19 quarantine [67]. This led to an increase in their body weight and elevated the obesity rate [67]. Moreover, the change in weight and BMI was strongly related to marital status, education level, place of residence, family size, family working members and employment status [67]. Another meta-analysis of a total of 74 studies with 3,213,776 participants showed that in the first year of the COVID-19 pandemic, slight but possibly clinically considerable elevations in body weight gain and BMI and a high incidence of obesity in children and adults were noted [68]. Moreover, outdoor activities were reduced throughout the COVID-19 pandemic, which was ascribed to social isolation and remaining at home [69]. In combination with the above, enhanced screen time due to online classes and more snacking resulted in a raised incidence of obesity and additional morbidities related to it in children and adolescents [69]. Nevertheless, the vast majority of currently available surveys examining the impact of COVID-19 on body weight concern younger populations such as children, adolescents or middle-aged adults. In contrast, the studies assessing the effects of COVID-19 on the elderly remain limited and mainly concern the non-healthy elderly. In this aspect, the present survey constitutes one of the few studies revealing that the COVID-19 pandemic could increase the frequency of being overweight or obese, and especially the occurrence of central obesity in healthy older adults.

Notably, it is well-documented that there is an interrelationship among depressive behavior, quality of life, cognition status and physical activity as well as nutrition status. In accordance with several available data sources, we found that the elderly had a two-fold greater probability of depression in the post-COVID-19 period than in the pre-COVID-19 period. Accordingly, a longitudinal survey of 2308 elderly Korean people, with a follow-up of two years, demonstrated that the COVID-19 pandemic resulted in elevated depressive symptomatology in the elderly. Moreover, it showed a double probability of the incidence of depression even in the euthymic elderly without signs of depressive symptomatology [70]. Moreover, fewer family gatherings, which were not associated with the risk of a depressive disorder before the pandemic, were associated with a doubled risk of a depressive disorder during the pandemic in older adults [70]. In addition, a retrospective survey, including 1004 outpatients (aged ≥60 years; mean age 70.8 ± 7.3 years), diagnosed 156 elderly people (15.5%) with depressive behavior [71]. In fact, the proportions of low, medium and major depression were 14.1%, 44.9% and 41.0%, respectively [71]. This study clearly indicated that COVID-19 could exert a direct or an indirect effect on depressive status in the elderly [71]. It also revealed a relation of COVID-19 infection history with a reduced occurrence of outdoor activities throughout the COVID-19 pandemia and depressive behavior in the elderly [71]. The English Longitudinal Study of Ageing also provided convincing data that depressive status among the elderly was substantially worsened during the COVID-19 pandemic [72]. Interestingly, this study found that mental health worsened significantly compared to the pre-pandemic period across all income groups of the older population, implying a limited role of income as a protective mechanism for mental health [72].

Furthermore, the National Health and Aging Trends Study in the USA conducted among 4548 elderly people demonstrated that dementia and poor activity participation resulted in an elevated frequency of depression symptoms and anxiety throughout the COVID-19 pandemic [73]. Poor activity participation was also correlated with a high risk of depressive symptoms and anxiety, and the association between dementia and worse mental health outcomes remained significant even after controlling for activity participation [73]. In this context, a Greek study including a convenience sample of 200 participants aged >65 years showed that during the pandemic, elderly people suffered from isolation, anxiety about COVID-19 and depressive symptoms that interrelated each other [74]. This study suggested developing Primary Health and Social Care policies. This aims at addressing the mental health problems of the older population that have been caused by the COVID-19 pandemic by developing their resilience, offering psychological support and promoting social connections [74]. In support of this view, a systematic review of 53 studies highlighted the most crucial features resulting in depression symptomatology in the elderly before the COVID-19 pandemic [75]. The most important factors were the sociodemographic parameters (i.e., being female), loneliness and low social support, restrictions in daily functioning, physical activity and neurocognitive damage [75]. In this aspect, a cross-sectional survey conducted through telephone interviews throughout the COVID-19 pandemic amongst 2077 Bangladeshi elderly people aged 60 years and above [76]. It showed a substantial rise in the incidence of depression in the 2021 study in comparison with the 2020 survey (47.2% vs. 40.3%, respectively) [76]. More to the point, this study showed that depression was significantly higher among participants without a partner, with a low monthly family income, living alone, feeling isolated, with poor memory/concentration, with non-communicable chronic conditions, overwhelmed by COVID-19, having difficulty earning or obtaining food during COVID-19 pandemic, communicating less frequently and needing extra care during the pandemic [76].

As far as physical activity is concerned, our enrolled older adult participants exhibited a 65% higher probability of low physical activity levels in the post-COVID-19 period in comparison with the pre-COVID-19 period. In this aspect, a retrospective survey conducted among 903 elderly people from urban areas showed that outside physical activity can exert a positive effect on the quality of life of elderly people [77]. Furthermore, physical activity was found to improve depression symptomatology in elderly people by enhancing self-efficacy and social support [78]. In an analysis of factors affecting depression in older adults in Korea, Kim and Park et al. found that cognitive decline was highly related with the development of depression in older adults. In addition, by participating in physical activity, they were able to maintain a healthy lifestyle and decrease the risk of cognitive decline, thereby preventing and reducing depression and improving health in older adults [79]. Moreover, a Greek community-based retrospective survey including 411 older adults with a mean age of 72.47 ± 6.89 years demonstrated that 43.5% (n = 179) of them reported a reduction in physical activity [80]. This was ascribed to the pandemic and community loneliness limitations [80]. This study suggested that decreased physical activity behavior in the elderly may slow down their probability of presenting frailty, sarcopenia and disability [80].

Accordingly, in a longitudinal study conducted among 500 community-dwelling older adults, the level of physical activity decreased because of the strict regulations during pandemic and did not recover rapidly in the post-COVID-19 period [81]. These findings reinforce our results concerning physical activity. It should be noted that physical activity can also reduce depression symptomatology in the elderly by enhancing self-efficacy through social support [78]. In a population-based survey, the COVID-19 pandemic led to a 72% reduction in physical activity levels and a 145% increase in physical inactivity in comparison with the post-COVID-19 period [82]. Social isolation, a systematic search for knowledge about COVID-19, worrying about the pandemia and COVID-19 infection were considered crucial parameters that resulted in the reduction in physical activity throughout the pandemia [82].

Another finding of our study concerns the fact that older adult participants showed an 87% higher likelihood of presenting worse cognitive impairment in the post-COVID-19 period in comparison with the pre-COVID-19 period. The above findings are in accordance with a retrospective survey conducted among 456 community-based elderly people with a mean age of 72.48 ± 6.84 years, contacted by telephone [83]. This study indicated that the COVID-19 pandemic exerted a negative effect on the mental health of the elderly people, especially those suffering from comorbidities or baseline functional dependence and those with previous depressive symptoms and cognitive damage [83]. In a study conducted among 18,813 Korean people, the cognitive function of the elderly declined throughout the COVID-19 pandemic, being related to poor community interactions due to the social isolation measures [84]. Additionally, in a multisite clinical trial conducted among 189 older adults (ages 65–89) during the pandemic, the participants reported low sleep quality, apparent physical health and functionality [85]. This study also indicated worse mental health, a small rise in depression and apathy symptoms, and decreased social engagement/apparent community support [85]. In addition, a longitudinal study has indicated that community isolation strategies because of the worldwide pandemia could result in higher probability of social loneliness and cognitive decline amongst the elderly [86]. For this purpose, this study suggested that the government and local communities should enhance their efforts to develop ways to connect older adults through the remainder of the pandemic and beyond [86].

In addition, a prospective cohort study analyzed 534 participants without subjective cognitive decline and found that 85 (15.9%) of them had novel subjective cognitive decline conditions one year after the beginning of COVID-19 pandemic [87]. In fact, walking and the need of personnel to advise were significant related parameters of novel subjective reduced cognition illnesses [87]. Another five-year (2016 to 2020) longitudinal survey was conducted among 1455 Korean older adults at the age of 72–84 years with a follow-up of two years [88]. This study demonstrated that the cognition function of participants weakened much more throughout the COVID-19 pandemic in comparison with prior to the pandemia, particularly concerning their memory function [88]. Malnutrition, depression, quality of life, low sleep quality and problems with deteriorating sleep were recognized as risk parameters for cognitive frailty amongst older adults in Thailand, which was ascribed to the COVID-19 pandemic [89]. Another study conducted in Belgium among older adults indicated that the presence of depressive and anxiety symptoms was related to the longer-term impact of the pandemic on wellbeing and subjective cognitive functioning [90].

Alarmingly, malnutrition during the Covid-pandemic constitutes a major concern, which is interrelated with depression, cognitive functionality and quality of life. Thus, it is not surprising that our enrolled participants showed a more than 2-fold greater risk of developing malnutrition in the post-COVID-19 period in comparison with the pre-COVID-19 period. Accordingly, amongst 150 consecutive patients hospitalized with COVID-19 pneumonia, 37 (24.3%) were malnourished, as detected by bioelectrical impedance vector analysis (BIVA), after a follow-up of two months from admission [91]. During the two months of follow-up, 10 (27%) undernourished patients and 13 (12%) non-undernourished patients needed invasive mechanical ventilation [91]. Additionally, 13 (35%) undernourished patients and 9 (8%) non-undernourished patients died [91]. The COVID-19 pandemic also led to a rise in the proportion of elderly people living in the community who were at risk of malnourishment. More to the point, the susceptible groups included individuals recovering at home from a mild-to-moderate COVID-19 infection, those discharged from hospital after serious infection and those who lived with prolonged periods of loneliness because of the public health measures aiming at reducing the expansion of the virus [91].

It is not surprising that the incidence of risk factors for malnourishment rose throughout the COVID-19 pandemic. These risk factors comprise diverse symptoms and effects of COVID-19, such as breathing difficulty, cough, inflammation, sarcopenia, anosmia, i.e., a decline in taste or smell, and the adverse effects of therapy. Moreover, public health infection prevention and control measures can considerably lower access to food and enhance social loneliness, thus negatively influencing individuals’ nutritional status [91]. In this context, in another study, 201 elderly people aged 65 and over were interviewed [92]. This study identified nutritional status as an essential factor during COVID-19 infection progression. Also, malnourishment was related to worse outcomes in hospitalized COVID-19 infected individuals [92]. Characteristically, a survey including 1230 COVID-19 survivors aged 18–86 attended a post-COVID-19 outpatient service [93]. In this study, the frequency of malnourishment was 22% at 4–5 months after acute disease. Notably, non-hospitalized individuals with acute COVID-19 disease showed a greater frequency of malnourishment than those requiring hospitalization (26% versus 19%) [93]. Malnourishment was also detected in 25% of COVID-19 survivors aged above 65 years in comparison with 21% of younger individuals [93]. After multivariable modification, the probability of malnutrition rose gradually and independently with increasing age [93].

Children and adolescents with high malnourishment risk assessed by STRONGkids at hospital admission were more likely to remain in the intensive care unit (ICU) [94]. They were also characterized by hospitalization longer than or equal to ten days [94]. In a prospective study of three hundred residents from three nursing homes, MNA test scores were decreased by 20% throughout the confinement [95]. This fact highlighted the necessity for preventive strategies to reduce the impact of future social and physical stressors on these vulnerable individuals [95]. Another longitudinal survey conducted among 1207 patients revealed that patients with serious COVID-19 symptomatology were more likely to be at risk of refeeding syndrome in comparison with those presenting low or modest COVID-19 symptomatology [96]. The patients presenting advanced COVID-19 disease progression were characterized by a 2.47 times higher frequency of refeeding syndrome than those with a low or modest COVID-19 infection progression [96]. Finally, a meta-analysis of 12 surveys showed that malnourishment or higher malnourishment risk raised the odds of in-hospital death by more than 3-fold [97]. The pooled incidence estimation of malnutrition or elevated malnourishment risk was 52.61%. Consequently, it has been considered that malnutrition may be a warning prognostic sign in hospitalized individuals diagnosed with COVID-19 [97].

The current survey exhibits several strengths since it included a quite representative sample of elderly people living in diverse geographical areas of our country. Therefore, its representativeness could be considered as relatively adequate. Thus, our results could be generalized to European populations of other origins. Additionally, this study constitutes one of the few studies that has explored the impact of COVID-19 pandemic on several interrelated factors such as sociodemographic and anthropometric characteristics, depressive behavior, quality of life, cognition status, physical activity and the nutritional status of the elderly. An additional strength of the present study is the fact that face-to-face interviews with the assigned older adults with experienced staff were accomplished to decrease memory biases. The systematic explanation instructions and the comprehensive presentation of the questionnaires, which were provided by the one-to-one interviews, could additionally decrease possible memory biases, enhancing the validity and reliability of the participants’ responses. Moreover, the ratio of male and female participants was practically 1:1 to minimize gender-depended impacts. In addition, the enrolled elderly were carefully chosen to contain a representative distribution of all age groupings over 65 years old. Another strength of the present survey concerns the use of validated, well-recognized and well-established questionnaires. A final strength of our study is that recruitment to the study took place quite a long time after the COVID-19 confinement, highlighting the persistent negative effect of the pandemic in older adults.

However, the current survey also exhibits some limitations, which could be considered for future studies. The cross-sectional methodology of the present survey reduces the likelihood of etiological conclusions and has the possibility of memory biases, particularly for self-reported questions, even though we conducted one-to one interviews with some participants. It should be emphasized that no conclusions concerning causality could be derived because of the cross-sectional methodology of the present survey. In this aspect, longitudinal studies should be performed to establish the causality effects of the COVID-19 pandemic on diverse aspects of the mental and physical health of older adults. Additionally, even though we used a comprehensive method to adjust for confounding factors, we recognize the probability of unmeasured confounding factors. Although we performed an adjustment for age, gender, nationality, education status, financial level, smoking behaviors, type of residence, employment status, parity and living status, and marital status, it remains probable that residual confounders could affect our findings. In this context, several other potential confounding factors related to mental health (e.g., anxiety, perceived stress, bipolar disorder, etc.) should be explored in future studies. Drug prescriptions constitute an additional probable confounder that must be taken into account in future studies, especially in nutritional intervention studies in which food ingredients may be interrelated with medicinal remedies. Even if our research is reasonably representative for our country, its results may not be generalized to other populations with probable different genetic backgrounds such as populations from Asia, America and Africa. Finally, it should be taken into consideration that the enrolled elderly of our study were 2–3 years older in the post-COVID-19 period compared with in the pre-COVID-19 period. This is a significant limitation of our study, as the COVID-19 pandemic and increasing age may lead to mixed effects that could affect the impact of the COVID-19 pandemic on the mental and physical health of the participants due to their increased age. However, this fact was unavoidable since we aimed to explore the same study population before and after the COVID-19 pandemic. In this aspect, age was included in the multivariate analysis and most of the mental and physical health parameters remained significant. Nevertheless, future studies could be performed using different but age-matched study populations before and after the COVID-19 pandemic to verify whether the present findings are independent of the age of participants.

## 5. Conclusions

The present survey constitutes one of the few surveys that has provided evidence that the COVID-19 pandemic has led to deleterious impacts on several aspects of the mental and physical health of the elderly, underlining that specific sociodemographic and anthropometric factors have also been affected negatively. Alarmingly, several deleterious effects of the COVID-19 pandemic appear to have persisted throughout the post-COVID-19 period, since a high prevalence of depression, cognitive impairment, physical inactivity, heavy smoking, obesity and malnutrition has been recorded, negatively affecting the daily quality of life of older adults. In view of the above considerations, urgent public policies and efficient strategies should be put in place to promote nutritional and psychological counseling and support for the persons who were significantly negatively influenced by the COVID-19 pandemic, and especially to older adults. Future prospective population-based and well-designed studies are strongly recommended to establish the causal effects of the COVID-19 pandemic in diverse aspects of the mental and physical health of the general population, with a special focus on older adults. Nutritional interventional studies are also needed to be performed to establish appropriate and efficient dietary patterns and foodstuffs that may prevent older adults from the harmful long-term effects of the COVID-19 pandemic. Traditional healthy dietary patterns such as the Mediterranean diet may be explored as protecting nutritional strategies against COVID-19 infection and as preventive policies to attenuate the long-term COVID-19 detrimental effects.

## Figures and Tables

**Table 1:** Comparative analysis of sociodemographic, anthropometric and lifestyle factors between pre-Covid and post-Covid periods.

Parameters	Pre-COVID Period	Post-COVID Period	*p*-Value
**Age (mean ± SD years)**			
	72.13 ± 8.1	75.22 ± 8.2	*p* = 0.0001
**Employment (n, %)**			
Employed	638 (15.3%)	431 (10.3%)	*p* = 0.0286
Unemployed	3524 (84.7%)	3731 (89.7%)	
**Type of residence (n, %)**			
Urban	2725 (65.5%)	2279 (54.8%)	*p* = 0.0187
Rural	1417 (34.5%)	1883 (45.2%)	
**Living status (n, %)**			
Living with others	3113 (74.8%)	2429 (58.4%)	*p* = 0.0087
Living alone	1049 (25.2%)	1733 (41.6%)	
**Educational level (n, %)**			
Primary education	1399 (33.6%)	1399 (33.6%)	*p* = 0.1931
Secondary education	1189 (28.6%)	1025 (24.6%)	
University studies	1574 (37.8%)	1738 (41.8%)	
**Family economic status (n, %)**			
Low	2347 (56.4%)	2575 (61.9%)	*p* = 0.0108
Medium	1248 (30.0%)	1157 (27.8%)	
High	567 (13.6%)	430 (10.3%)	
**Smoking habits (n, %)**			*p* = 0.0031
Smokers	687 (16.5%)	1441 (34.6%)	
Never smoked	3475 (83.5%)	2721 (65.4%)	
**BMI (mean ± SD Kg/m^2^)**	27.3 ± 4.1	28.9 ± 4.2	*p* = 0.0005
**BMI status (n, %)**			
Normal Weight	2892 (69.5%)	2324 (55.8%)	*p* = 0.0002
Overweight	968 (23.2%)	1247 (30.0%)	
Obese	302 (7.3%)	591 (14.3%)	
**WHR (n, %)**			*p* = 0.0107
Low	3115 (74.8%)	2819 (67.7%)	
Medium	703 (16.9%)	912 (21.9%)	
High	344 (8.3%)	431 (10.4%)	
**GDS (mean ± SD)**	12.2 ± 4.7	14.5 ± 4.3	*p* ˂ 0.0001
**Depression (n, %)**			*p* = 0.0012
No			*p* = 0.0005
Yes			
**HRQOL score (mean ± SD)**	51.1 ± 11.3	53.4 ± 11.2	*p* = 0.0152
**MMSE score (mean ± SD)**	24.4 ± 5.1	22.3 ± 5.2	*p* = 0.0001
**Cognitive status (n, %)**			*p* = 0.0008
No cognitive impairment	2830 (68.0%)	2174 (52.2%)	
Mild cognitive impairment	750 (18.0%)	1208 (29.0%)	
Moderate/severe cognitive impairment	582 (14.0%)	780 (18.7%)	
**IPAQ status (n, %)**			*p* = 0.0028
Low	1639 (39.4%)	2221 (53.4%)	
Medium	1321 (31.7%)	1252 (30.1%)	
High	1202 (28.9)	689 (16.6%)	
**MNA (mean ± SD)**	23.8 ± 4.2	22.1 ± 4.5	*p* = 0.0001
**Nutritional status (n, %)**			*p* ˂ 0.0001
Malnutrition	405 (9.7%)	601 (14.4%)	
At risk of malnutrition	1268 (30.5%)	1985 (47.7%)	
Normal nutrition	2489 (59.8%)	1576 (37.9%)	

**Table 2:** Multivariate analysis of the comparison of pre- and post-COVID-19 periods concerning sociodemographic, anthropometric and lifestyle factors.

Patients’ Characteristics	Pre-Covid Period vs. Post-Covid Period	
	RR * (95% CI **)	*p*-Value
**Age** (Below/Over mean value)	1.63 (1.34–1.91)	*p* = 0.0252
**Employment** (Employed/Unemployed)	1.12 (0.71–1.69)	*p* = 0.0901
**Type of residence** (Urban/Rural)	1.21 (0.80–1.65)	*p* = 0.1108
**Living status** (Living with others/Living alone)	1.18 (0.81–1.72)	*p* = 0.1276
**Educational level** (Primary and secondary education/University studies)	1.03 (0.45–1.66)	*p* = 0.4581
**Family economic status** (High/Moderate and Low)	1.21 (0.72–1.85)	*p* = 0.4675
**Smoking habits** (No/Yes)	1.71 (1.49–2.03)	*p* = 0.0210
**BMI status** (Normal weight/Overweight + Obesity)	1.88 (1.68–2.09)	*p* = 0.0072
**WHR** (Low/Medium +High)	1.79 (1.52–2.09)	*p* = 0.0202
**Depression** (No/Yes)	2.11 (1.94–2.23)	*p* = 0.0001
**HRQOL** (Over/Below mean value)	1.95 (1.69–2.21)	*p* = 0.0372
**Cognitive status** (Normal cognitive status/Mild + Moderate + Severe cognitive impairment)	2.23 (1.99–2.44)	*p* = 0.0042
**IPAQ** (High and Moderate/Low)	1.87 (1.56–2.18)	*p* = 0.0193
**MNA** (Low/Medium + High)	2.34 (2.15–2.56)	*p* = 0.0005

* Relative risk: OR. ** CI: Confidence interval.

## Data Availability

Data are available upon request to the corresponding author.
